# Resistin upregulates chemokine production by fibroblast-like synoviocytes from patients with rheumatoid arthritis

**DOI:** 10.1186/s13075-017-1472-0

**Published:** 2017-12-01

**Authors:** Hiroshi Sato, Sei Muraoka, Natsuko Kusunoki, Shotaro Masuoka, Soichi Yamada, Hideaki Ogasawara, Toshio Imai, Yoshikiyo Akasaka, Naobumi Tochigi, Hiroshi Takahashi, Kazuaki Tsuchiya, Shinichi Kawai, Toshihiro Nanki

**Affiliations:** 1 Department of Internal Medicine, Graduate School of Medicine, Toho University, Tokyo, Japan; 2 Division of Rheumatology, Department of Internal Medicine, Toho University School of Medicine, 6-11-1 Omori-Nishi, Ota-ku, Tokyo, 143-8541 Japan; 3 KAN Research Institute Inc, 6-8-2 Minatojima-minamimachi, Chuo-Ku, Kobe, 650-0047 Japan; 4 Unit of Regenerative Diseases Research, Division of Research Promotion and Development, Advanced Medical Research Center, Toho University Graduate School of Medicine, Tokyo, Japan; 5 Department of Surgical Pathology, Toho University School of Medicine, Tokyo, Japan; 6 Department of Orthopedic Surgery, Toho University School of Medicine, Tokyo, Japan; 7 Department of Inflammation and Pain Control Research, Toho University School of Medicine, Tokyo, Japan

**Keywords:** Resistin, Adenylate cyclase-associated protein 1, Rheumatoid arthritis, Chemokine, Fibroblast-like synoviocytes, RNA sequencing

## Abstract

**Background:**

Adipokines are bioactive hormones secreted by adipose tissues. Resistin, an adipokine, plays important roles in the regulation of insulin resistance and inflammation. Resistin levels are known to be increased in the serum and synovial fluid of rheumatoid arthritis (RA) patients. However, the pathogenic role of resistin in RA has not yet been elucidated.

**Methods:**

The expression of resistin and adenylate cyclase-associated protein 1 (CAP1), a receptor for resistin, was examined immunohistochemically in synovial tissue. CAP1 expression in in vitro cultured fibroblast-like synoviocytes (FLSs) was assessed with a reverse transcription-polymerase chain reaction (PCR) and western blotting. The gene expression of resistin-stimulated FLSs was evaluated by RNA sequencing (RNA-Seq) and quantitative real-time PCR. Concentrations of chemokine (C-X-C motif) ligand (CXCL) 8, chemokine (C-C motif) ligand (CCL) 2, interleukin (IL)-1β, IL-6 and IL-32 in culture supernatants were measured by enzyme-linked immunosorbent assay. Small interfering RNA (siRNA) for CAP1 was transfected into FLSs in order to examine inhibitory effects.

**Results:**

The expression of resistin and CAP1 in synovial tissue was stronger in RA than in osteoarthritis (OA). Resistin was expressed by macrophages in the RA synovium, while CAP1 was expressed by macrophages, FLSs and endothelial cells. In vitro cultured RA FLSs also expressed CAP1. RNA-Seq revealed that the expression levels of 18 molecules were more than twofold higher in resistin-stimulated FLSs than in unstimulated FLSs. Seven chemokines, CXCL1, CXCL2, CXCL3, CXCL5, CXCL6, CXCL8, and CCL2, were included among the 18 molecules. Increases induced in the expression of CXCL1, CXCL8, and CCL2 by the resistin stimulation were confirmed by real-time PCR. The stimulation with resistin increased the protein levels of CXCL8 and CCL2 produced by RA FLSs, and the upregulated expression of CXCL8 was inhibited by the abrogation of CAP1 by siRNA for CAP1. Production of IL-6 by FLSs was also increased by resistin. Expression of IL-1β and IL-32 was not detected by ELISA.

**Conclusions:**

Resistin contributes to the pathogenesis of RA by increasing chemokine production by FLSs via CAP1 in synovial tissue.

## Background

Adipokines are bioactive hormones secreted by adipose tissues. More than 600 adipokines have been identified to date (e.g. adiponectin, leptin, tumor necrosis factor α, interleukin (IL)-1, IL-6, apelin, visfatin, and resistin) [1, 2]. Resistin was discovered as a protein secreted by differentiated 3T3-L1 cells, and its expression was found to be downregulated by treatment with thiazolidinedione rosiglitazone [3]. In mice, resistin is mainly expressed by mature adipocytes in white adipose tissue. In contrast, resistin in humans is mainly expressed by monocytes and macrophages and less so by adipocytes [4]. Therefore, resistin may contribute not only to insulin resistance, but also inflammation.

Rheumatoid arthritis (RA) is characterized by chronic polyarthritis. Inflammatory mediators, such as cytokines and chemokines, contribute to the pathogenesis of RA. The immunomodulatory properties of adipokines in RA have been evaluated [5]. Adiponectin enhances the production of proinflammatory factors (IL-6 and chemokine (C-X-C motif) ligand (CXCL) 8), vascular endothelial growth factor, and matrix metalloproteinases (MMPs) by fibroblast-like synoviocytes (FLSs) [6–8]. Previous meta-analyses revealed that serum resistin levels are higher in patients with RA than in healthy controls [9]. Furthermore, we previously demonstrated that the serum level of resistin is positively associated with serum C-reactive protein levels in patients with RA [10], while another group showed that the concentration of resistin is elevated in the synovial fluid in RA [11]. However, the pathogenic role of resistin in RA has not yet been elucidated.

In the present study, we examined the stimulatory effects of resistin on FLSs from patients with RA using RNA sequencing (RNA-Seq). We found that the expression of chemokines was increased in resistin-stimulated FLSs.

## Methods

### Samples

Synovial tissues were obtained from patients with RA and with osteoarthritis (OA) who underwent total knee or hip replacement. FLSs were prepared from synovial tissues as described previously [12]. FLSs from RA synovial tissues were also obtained from the Japanese Collection of Research Bioresources Cell Bank. The experimental protocol was approved in advance by the Ethics Committees of Toho Medical Center Omori Hospital (M16020) and the Ethics Committees of the Faculty of Medicine, Toho University (27060, 2703024007).

### Immunohistochemical assessment

Synovial tissues were fixed with freshly prepared 4% (v/v) paraformaldehyde in Tris-buffered saline. Sections (3 μm) were immersed in ethanol containing 3% (v/v) H_2_O_2_ for 30 min to block endogenous peroxidase activity. Sections were incubated with protein block serum-free (Agilent Technologies) for 30 min to block non-specific binding. Sections were subsequently incubated at 4 °C overnight with a rabbit anti-resistin polyclonal antibody (pAb) (Bioss Antibodies), rabbit anti-adenylyl cyclase-associated protein 1 (CAP1) monoclonal antibody (mAb) (EPR8339(B); Abcam), or isotype control (Agilent Technologies) as a primary antibody. Expression was detected using an EnVision + kit™ (Agilent Technologies) and counterstained with hematoxylin.

Non-specific binding was blocked with protein block serum-free for immunofluorescence double staining, and sections were incubated at 4 °C overnight with rabbit anti-resistin pAb or CAP1 mAb. Samples were subsequently incubated at room temperature with biotinylated anti-rabbit IgG for 40 min followed by Fluorescein Avidin D (Vector laboratories) for 20 min. Samples were incubated with mouse anti-CD68 mAb (KP1; Abcam), mouse anti-cadherin-11 mAb (16A; Acris Antibodies), or mouse anti-von Willebrand factor (vWF) mAb (F8/86; Agilent Technologies) and then with a Texas Red® horse anti-mouse IgG antibody at room temperature for 20 min. A nuclear stain was performed with 4′, 6-diamidino-2-phenylindole. Slides were examined using the BX61 (Olympus). To determine the percentages of CAP1-expressing cells, the number of CAP1-positive cells in the lining layer, and in the CD68-positive or cadherin-11-positive cells was counted under fluorescence microscope.

### RNA extraction

FLSs were seeded in Roswell Park Memorial Institute (RPMI) 1640 medium containing 10% fetal bovine serum (FBS) in 10-cm dishes (1 × 10^6^ cells/dish), and were then incubated with 1000 ng/ml resistin (PeproTech) for 18 h. Total RNA was isolated from FLSs using TRIzol® (Invitrogen) according to the manufacturer’s instructions. RNA samples were digested with an RNase-free DNase set (Qiagen) to remove genomic DNA and further purified using the RNeasy kit (Qiagen). The quality of RNA samples was examined by the Agilent 2100 Bioanalyzer (Agilent Technologies) using RNA 6000 NanoChips. RNA samples with an RNA integrity number higher than 7 were used in further analyses, including a RNA-Seq and real-time polymerase chain reaction (PCR).

### RNA-Seq transcriptome analysis

The RNA-Seq library was prepared using the SureSelect Strand-Specific RNA Library Prep Kit (Agilent Technologies) in accordance with the manufacturer’s instructions optimized to Illumina Multiplexed Sequencing. After purification of the amplified libraries, the DNA quality of products was assessed using the 2100 Bioanalyzer DNA 1000 Assay. Paired-end sequencing of the RNA-seq libraries was performed using an Illumina MiSeq system (Illumina; 2 × 75 bases paired-end run). FASTQ files were imported into CLC Genomics Workbench v9.01 software (CLC bio) for post-processing and data analysis. Sequences were trimmed based on the FASTQC report and mapped onto annotated human genes with support from reference human genome (hg19). Data were normalized by total reads per million and analyzed for differential gene expression empirical analysis of differential gene expression subroutine based on reads per kilobase of transcript per million reads mapped (RPKM).

### Quantitative real-time PCR

Total RNA samples were reverse transcribed into cDNA using random primers and an RNA PCR Kit (AMV) Ver.3.0 (Takara Bio Inc.). CXCL1, CXCL8, and chemokine (C-C motif) ligand (CCL) 2 levels were measured using the Power SYBR Green PCR Master Mix (Applied Biosystems). The following primers were used for analyses: 5′-TGC AGG GAA TTC ACC CCA AG-3′ and 5′-CAG GGC CTC CTT CAG GAA CA-3′ for CXCL1; 5′-ACT CCA AAC CTT TCC ACC CCA-3′ and 5′-TTT CCT TGG GGT CCA GAC AGA-3′ for CXCL8; 5′-CTT CTG TGC CTG CTG CTC AT-3′ and 5′-CGG AGT TTG GGT TTG CTT GTC-3′ for CCL2; 5′-GAA GGT GAA GGT CGG AGT CA-3′ and 5′-GAG GTC AAT GAA GGG GTC AT-3′ for glyceraldehyde-3-phosphate dehydrogenase (GAPDH). The Prism 7500 Fast Real-time PCR system (Applied Biosystems) was used for analyses, and the mRNA levels of the genes tested were represented as relative values to the expression level of GAPDH.

### Reverse transcription (RT)-PCR

Total RNA was extracted with an RNeasy Mini kit (Qiagen) from the cultured FLSs of patients with RA. RT was performed using a SuperScript first-strand synthesis system for RT-PCR according to the recommendations of the manufacturer (Invitrogen) with 1 μg of total RNA from FLSs. Equal amounts of each RT product were amplified by PCR with HotStarTaq® DNA polymerase (Qiagen). The primer sequences and numbers of base pairs (bp) were as follows: for CAP1 (118 bp), 5′-AGG CAT TTG ACT CGC TGC TTG and 5′- TCG CTC CAA CTT CAA ACC TGT G; and for GAPDH (598 bp), 5′- CCA CCC ATG GCA AAT TCC ATG GCA and 5′-TCT AGA CGG CAG GTC AGG TCC ACC. After initial denaturation at 95 °C for 15 min, PCR involved amplification for 32 cycles at 95 °C for 30 s, at 56 °C for 30 s, and at 72 °C for 45 s, followed by elongation at 72 °C for 5 min. Amplified DNA fragments were resolved by electrophoresis on a 2% agarose gel, and were detected under ultraviolet light using LAS-3000 (Fujifilm) after staining the gel with ethidium bromide.

### Western blot analysis

The western blotting procedure was previously described [13]. Membranes were incubated with rabbit anti-CAP1 mAb (EPR8339(B); Abcam) or rabbit anti-GAPDH pAb (Santa Cruz Biotechnology), with a dilution of 1:1000 (CAP1) and 1:100 (GAPDH), the secondary antibody (horseradish peroxidase-conjugated goat anti-rabbit antibody) was added (at a dilution of 1:2000), and an incubation was performed for 3 h using the iBind Flex Western System (Thermo Fisher Scientific). Protein bands were detected with the enhanced Novex® ECL Chemiluminescent Substrate Reagent Kit (Invitrogen) using LAS-3000 (Fujifilm).

### Enzyme-linked immunosorbent assay (ELISA)

FLSs were cultured overnight in 96-well plates (2 × 10^4^ cells/well) and then incubated with recombinant human resistin (0, 10, 100, or 1000 ng/ml; PeproTech) at 37 °C for 24 h in RPMI1640 medium containing 1% FBS. Concentrations of CXCL8, CCL2, IL-1β, IL-6, and IL-32 in culture supernatants were assessed using the ELISA kit (R&D Systems), according to the instructions of the manufacturer.

### Signaling pathway of resistin via CAP1

Stealth RNAi™ small interfering RNA (siRNA) targeting CAP1 and negative control siRNA were purchased from Thermo Fisher Scientific. Lipofectamine® RNAiMAX reagent (Thermo Fisher Scientific) was used to formulate transfecting siRNAs. FLSs were transfected with siRNA at 37 °C for 48 h, and cells were then treated with resistin (PeproTech) for another 24 h. CXCL8 concentrations in the culture supernatant were assessed by ELISA.

### Statistical analysis

Results are expressed as the mean +/- standard error (SE). Statistical analyses were performed using StatFlex software (ver. 6; ARTEC). The production of CXCL8, CCL2, and IL-6 was analyzed by analysis of variance using Dunnett’s test. The paired *t* test was applied to compare CXCL8 production between control siRNA-transfected and CAP1 siRNA-transfected cells. In all analyses, *p* < 0.05 was considered to indicate significance.

## Results

### Expression of resistin and CAP1 in synovial tissues of RA

**Fig. 1:**
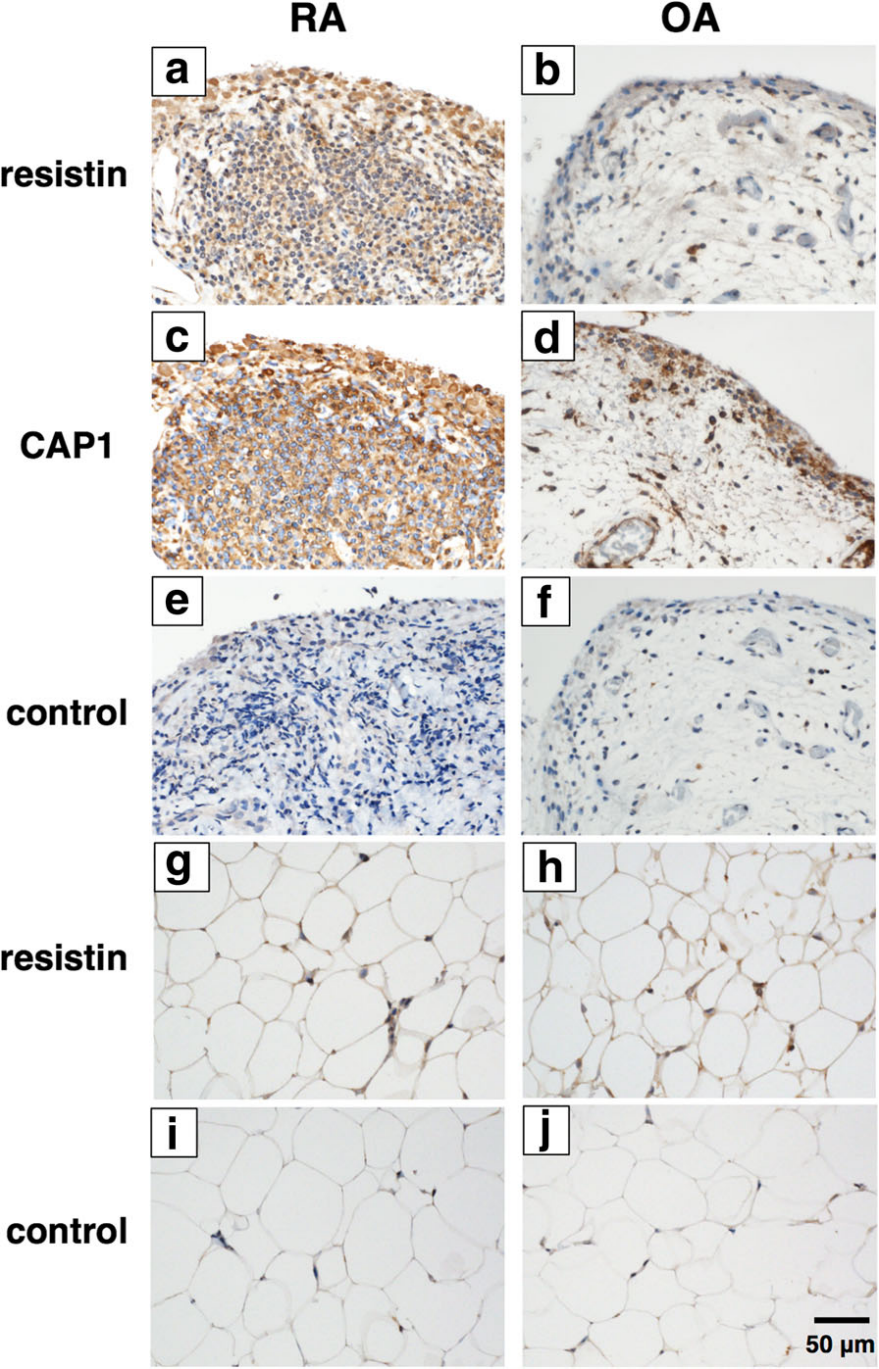
Expression of resistin and adenylate cyclase-associated protein 1 (CAP1) in the synovial tissue in rheumatoid arthritis (RA). Synovial tissue from RA (**a**, **c**, **e**, **g** and **i**) or osteoarthritis (OA) (**b**, **d**, **f**, **h** and **j**) was stained with rabbit anti-resistin polyclonal antibody (**a**, **b**, **g** and **h**), rabbit anti-CAP1 monoclonal antibody (**c** and **d**), or control antibody (**e**, **f**, **i** and **j**). All sections were counterstained with hematoxylin. The representative figures of three tissue sections from RA and three from OA are depicted

We immunohistochemically investigated the expression of resistin and CAP1 in synovial tissues harvested from patients with RA and with OA. Resistin was strongly expressed in the synovial lining and sub-lining cells of synovial tissue in RA (Fig. 1a), while resistin expression was minimal in the synovium in OA (Fig. 1b). On the other hand, the expression of CAP1 was observed in the lining and sub-lining cells of the synovium in RA (Fig. 1c), while CAP1 was expressed in the lining cells of the synovium in OA (Fig. 1d). In fat tissue around the synovial tissue, resisitin was weakly expressed in RA and OA (Fig. 1g and h).

**Fig. 2:**
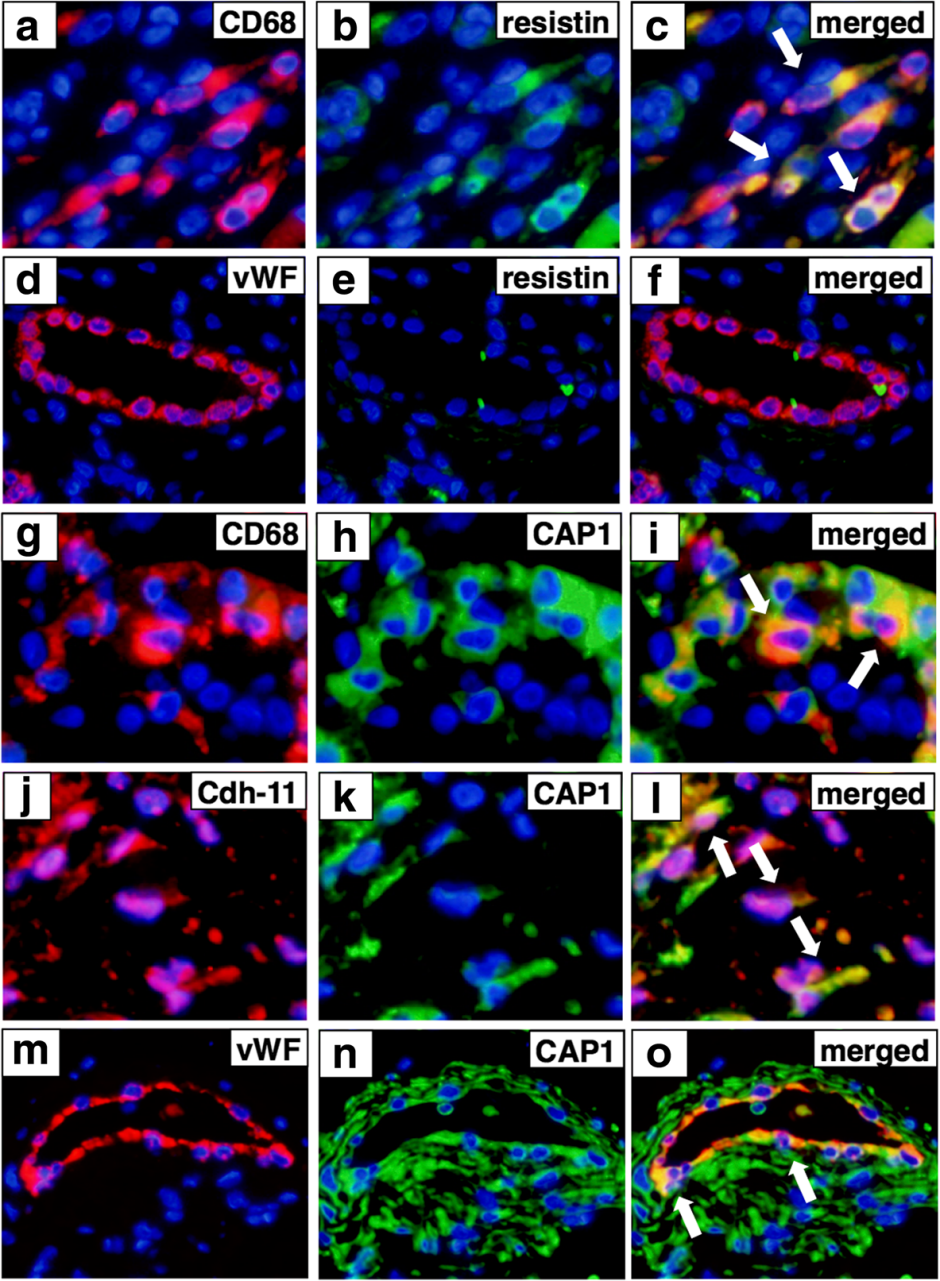
Resistin-expressing and adenylate cyclase-associated protein 1 (CAP1)-expressing cells in synovial tissues in rheumatoid arthritis (RA). Sections of synovial tissue from RA were double-stained with resistin, and CD68 or von Willebrand factor (vWF) (CD68 (**a**); resistin (**b**); merge of **a** with **b** (**c**); vWF (**d**); resistin (**e**); merge of **d** with **e** (**f**)), and CAP1, and CD68, cadherin-11 or vWF (CD68 (**g**); CAP1 (**h**); merge of **g** with **h** (**i**); cadherin-11 (**j**); CAP1 (**k**); merge of **j** with **k** (**l**); vWF (**m**); CAP1 (**n**); merge of **m** with **n** (**o**)). Arrows indicate double-positive cells in the merged image. Cdh-11, cadherin-11

We performed double immunohistochemical assessment to identify resistin-expressing and CAP1-expressing cells in the synovium in RA. As shown in Fig. 2, resistin was expressed by CD68^+^ macrophages (Fig. 2a-c). However, resistin was not expressed by vWF^+^ endothelial cells (Fig. 2d-f). CAP1 was expressed by CD68^+^ macrophages (Fig. 2g-i) and also by cadherin-11^+^ FLSs (Fig. 2j-l) and vWF^+^ endothelial cells (Fig. 2m-o) in synovial tissues in RA. The percentage of CAP1-positive cells was 94% (187/200) in the lining layer. The frequency of CAP1-positive cells in CD68^+^ cells was 97% (97/100) in the lining layer and 95% (95/100) in the sub-lining layer. The percentage of CAP1-positive cells in cadherin-11^+^ cells was 95% (95/100) in the lining layer and 81% (81/100) in the sub-lining layer.

**Fig. 3:**
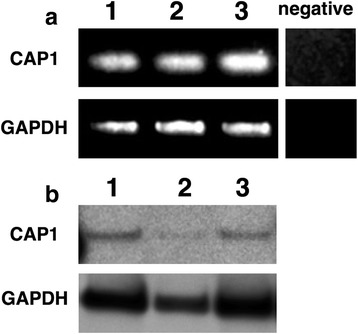
Expression of adenylate cyclase-associated protein 1 (CAP1), the receptor for resistin, in in vitro cultured fibroblast-like synoviocytes (FLSs). The expression of CAP1 mRNA was analyzed by RT-PCR in FLSs from three patients with RA (**a**). The expression of the CAP1 protein in three FLSs was examined by western blotting (**b**). negative, PCR without cDNA. GAPDH, glyceraldehyde-3-phosphate dehydrogenase

CAP1 expression in cultured FLSs established from synovial tissue in RA was evaluated with RT-PCR and western blotting. The mRNA and protein expression of CAP1 was also observed in in vitro cultured FLSs (Fig. 3).

### Stimulatory effects of resistin on FLSs in RA

We examined the stimulatory effects of resistin on FLSs in vitro. FLSs were incubated with 1000 ng/ml resistin for 18 h. Total RNA was extracted from the cells, and complementary DNA (cDNA) was synthesized. The nucleotide sequence of cDNA was analyzed by next-generation sequencing and expression levels were compared between unstimulated and resistin-stimulated FLSs. As shown in Table 1, the expression levels of 18 molecules were more than twofold higher in all three lots of resistin-stimulated FLSs than in unstimulated FLSs. Seven chemokines, CXCL1, CXCL2, CXCL3, CXCL5, CXCL6, CXCL8, and CCL2 were included among the 18 molecules.

**Table 1:** Increased gene expression by resistin-stimulated RA FLSs

Gene	Description	Fold change (resistin/not stimulated)				RPKM						Ensembl gene ID
						Lot 1		Lot 2		Lot 3		
		Lot 1	Lot 2	Lot 3	Mean	Non	RS	Non	RS	Non	RS	
*CXCL5*	*C-X-C motif chemokine ligand 5*	3.51	31.88	3.40	*12.93*	0.088	0.310	0.151	4.822	0.245	0.832	ENSG00000163735
*CXCL6*	*C-X-C motif chemokine ligand 6*	7.76	20.32	3.76	*10.61*	0.205	1.594	0.106	2.144	0.821	3.089	ENSG00000124875
*IL34*	*Interleukin 34*	17.56	5.63	2.37	*8.52*	0.033	0.588	0.172	0.967	0.309	0.732	ENSG00000157368
*CXCL1*	*C-X-C motif chemokine ligand 1*	3.13	18.39	3.63	*8.39*	2.538	7.952	1.066	19.609	3.991	14.506	ENSG00000163739
*CXCL8*	*C-X-C motif chemokine ligand 8*	5.07	10.25	4.21	*6.51*	0.592	3.002	1.384	14.183	1.152	4.853	ENSG00000169429
*BIRC3*	*Baculoviral IAP repeat containing 3*	3.44	7.06	6.42	*5.64*	0.230	0.789	0.195	1.376	0.129	0.828	ENSG00000023445
*IL1B*	*Interleukin 1 beta*	2.20	10.47	4.12	*5.59*	0.097	0.212	0.149	1.558	0.134	0.550	ENSG00000125538
*AFP*	*Alpha fetoprotein*	4.39	6.56	2.57	*4.51*	0.023	0.101	0.024	0.155	0.106	0.272	ENSG00000081051
*SOD2*	*Superoxide dismutase 2*	3.27	5.33	3.63	*4.08*	4.587	15.002	4.327	23.048	5.692	20.663	ENSG00000112096
*CCL2*	*C-C motif chemokine ligand 2*	2.31	5.80	2.89	*3.66*	20.170	46.526	23.381	135.522	16.826	48.585	ENSG00000108691
*ANXA8L1*	*Annexin A8 like 1*	2.63	3.19	4.94	*3.59*	0.035	0.093	0.180	0.575	0.032	0.160	ENSG00000264230
*SLC5A2*	*Solute carrier family 5 member 2*	3.51	2.11	4.94	*3.52*	0.017	0.059	0.069	0.145	0.015	0.076	ENSG00000140675
*CXCL3*	*C-X-C motif chemokine ligand 3*	3.95	3.09	2.63	*3.23*	0.270	1.065	0.346	1.071	0.466	1.228	ENSG00000163734
*ZNF296*	*Zinc finger protein 296*	2.05	3.75	2.96	*2.92*	0.129	0.263	0.088	0.330	0.119	0.352	ENSG00000170684
*ICAM1*	*Intercellular adhesion molecule 1*	2.29	3.56	2.43	*2.76*	2.380	5.455	2.590	9.227	1.935	4.705	ENSG00000090339
*IL32*	*Interleukin 32*	3.25	2.33	2.39	*2.66*	0.690	2.245	3.020	7.042	1.061	2.539	ENSG00000008517
*LY75*	*Lymphocyte antigen 75*	2.63	2.81	2.30	*2.58*	0.016	0.042	0.024	0.069	0.044	0.101	ENSG00000054219
*CXCL2*	*C-X-C motif chemokine ligand 2*	2.07	2.29	2.33	*2.23*	0.698	1.444	0.819	1.872	1.011	2.361	ENSG00000081041

*FLSs* fibroblast-like synoviocytes, *RA* rheumatoid arthritis, *RPKM* reads per kilobase of transcript per million reads mapped, *R*S resistin-stimulated, *No*n non-stimulated. FLSs from synovial tissue in RA were incubated with 1000 ng/ml resistin for 18 h. mRNA expression was analyzed by next-generation sequencing. Genes with expression levels that were more than twofold higher in resistin-stimulated FLSs than in unstimulated FLSs are shown

**Fig. 4:**
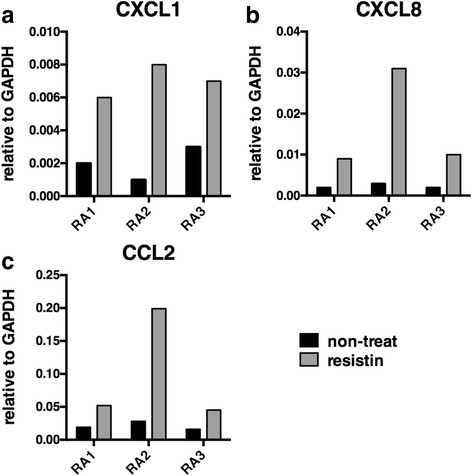
Increased chemokine expression by fibroblast-like synoviocytes (FLSs) from patients with rheumatoid arthritis (RA) with resistin stimulation. FLSs were incubated with 1000 ng/ml resistin for 18 h. The mRNA expression of CXCL1 (**a**), CXCL8 (**b**), and CCL2 (**c**) by FLSs from three patients with RA was measured using quantitative real-time reverse transcription PCR. GAPDH, glyceraldehyde-3-phosphate dehydrogenase

We also analyzed CXCL1, CXCL8, and CCL2 expression by quantitative real-time RT-PCR using three lots of FLSs from patients with RA. The expression of CXCL1, CXCL8, and CCL2 increased in all three lots of FLSs following the resistin stimulation (Fig. 4).

### Chemokine production by the resistin-CAP1 pathway

**Fig. 5:**
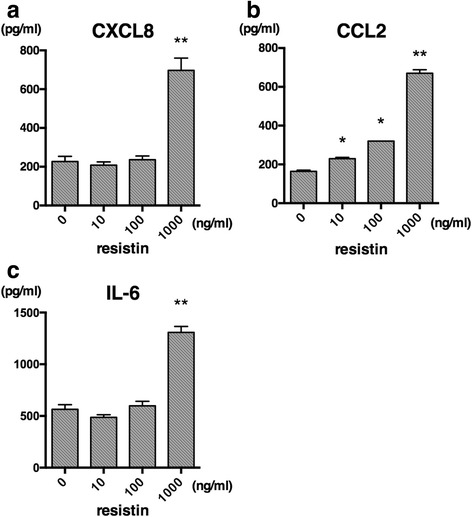
CXCL8, CCL2 and IL-6 expression by resistin-stimulated fibroblast-like synoviocytes (FLSs) from patients with rheumatoid arthritis (RA). FLSs from patients with RA were incubated with resistin (10–1000 ng/ml) for 24 h, and the concentrations of CXCL8 (**a**), CCL2 (**b**), and IL-6 (**c**) in the culture supernatant were measured by ELISA. Data are the mean +/- SE for one of three independent experiments analyzed in triplicate: *p < 0.05, **p < 0.01, versus no stimulation

We examined the protein levels of chemokine expression by resistin-stimulated FLSs in vitro. FLSs were incubated with various concentrations of resistin for 24 h. The concentrations of CXCL8 and CCL2, which were observed as upregulated chemokines by RNA-seq and real-time RT-PCR, were assessed in culture supernatants using ELISA kits. The CXCL8 level was significantly increased by the stimulation with resistin (Fig. 5a). CCL2 expression was dose-dependently increased by resistin (Fig. 5b). We also analyzed expression of IL-1β and IL-32, which were identified as upregulated cytokines by RNA-seq (Table 1), and IL-6, which was identified as a slightly upregulated cytokine by RNA-seq (fold change 1.969, 3.721, and 1.997 in each lot). IL-1β and IL-32 were not detected by ELISA with or without stimulation with resistin. The concentration of IL-6 was increased by resistin (Fig. 5c).

**Fig. 6:**
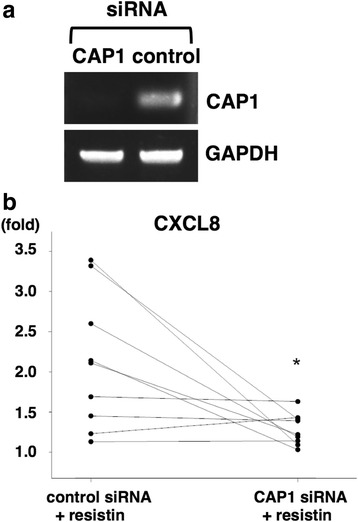
Inhibition of the resistin stimulation by adenylate cyclase-associated protein 1 (CAP1) abrogation. Fibroblast-like synoviocytes (FLSs) from patients with rheumatoid arthritis (RA) were pretreated by transfection with CAP1 siRNA or negative control siRNA. The expression of CAP1 mRNA was examined by RT-PCR (**a**). RA FLSs were then incubated with resistin (1000 ng/ml) for 24 h. CXCL8 levels in the culture supernatant were examined by ELISA (**b**). n = 9, *p < 0.05 versus control siRNA. GAPDH, glyceraldehyde-3-phosphate dehydrogenase

In order to verify the involvement of CAP1 in the resistin stimulation, siRNA for CAP1 was transfected into RA FLSs. The FLSs were pretreated with CAP1 siRNA or control siRNA. The transfection of CAP1 siRNA significantly decreased CAP1 expression from that with the transfection of control siRNA (Fig. 6a). Resistin-induced CXCL8 production by FLSs was significantly inhibited by the abrogation of CAP1 by siRNA (Fig. 6b). These results indicate that the resistin-CAP1 pathway contributes to chemokine production by RA FLSs.

## Discussion

The purpose of the present study was to elucidate the role of resistin in the pathogenesis of RA. We found that the expression of resistin was increased in synovial tissue in RA, and stimulation with resistin enhanced the production of various chemokines by FLSs via CAP1. These results suggest that resistin contributes to inflammatory cell infiltration into synovial tissue in RA through chemokine production by FLSs.

We previously reported that serum resistin levels are associated with C-reactive protein levels [10]. In the present study, we showed that resistin was strongly expressed in macrophages in synovial tissue in RA, which is consistent with previous findings [11]. A recent study reported that CAP1 is a functional receptor for resistin in THP-1 cells [14]. We found that CAP1 is more abundantly expressed in synovial tissue in RA than in OA. CAP1 is expressed by macrophages, FLSs and endothelial cells in synovial tissue in RA, and in in vitro cultured FLSs. These results suggest that resistin, an adipokine, stimulates CAP1-expressing macrophages, FLSs and endothelial cells in synovial tissue in RA. CAP1 expression in HP-AEpiC cells is decreased by treatment with matrix metalloproteinase 9 (MMP-9) [15]. On the other hand, we examined CAP1 expression on FLSs treated with TNF-α, IL-1β and resistin. These stimulations did not alter CAP1 expression significantly (data not shown). Regulation of CAP1 expression in the RA synovial cells has not been elucidated.

Toll-like receptor 4 (TLR4), decorin and receptor tyrosine kinase like orphan receptor 1 (ROR1) were reported as putative receptors for resistin [16–18]. Lee et al. [14] identified CAP1 as a functional receptor for resistin on monocytes. Abrogation of CAP1 inhibited production of inflammatory cytokines and cellular migration by stimulation with resistin. However, abrogation of TLR4, decorin and ROR1 had little effect on the resistin stimulation. Based on the results, we thought that CAP1 is a functional receptor for resistin. However, the function of TLR4, decorin and ROR1 for resistin on FLSs has not been clarified yet. We have found that at least TLR4 was expressed on FLSs. Therefore, further study is needed to show the function of the three putative receptors against resistin stimulation on FLSs.

Using RNA-seq, we found that the stimulation with resistin enhanced the expression of 18 genes by FLSs in vitro. Seven chemokines, CXCL1, CXCL2, CXCL3, CXCL5, CXCL6, CXCL8, and CCL2, were included. Furthermore, six out of the seven chemokines were C-X-C motif chemokines. Several C-X-C motif chemokines (CXCL1, CXCL2, CXCL3, CXCL5, CXCL6, CXCL7, and CXCL8) contain an ELR motif (Glu-Leu-Arg) at the NH2 terminus [19]. These ELR^+^ C-X-C motif chemokines could promote angiogenesis [20]. Chemokines upregulated by resistin stimulation in the present study were mostly ELR^+^ C-X-C motif chemokines. Therefore, resistin may be involved in angiogenesis and inflammatory cell accumulation, in the synovial tissue in RA via ELR^+^ C-X-C motif chemokine production.

In the present study, we also demonstrated that stimulation with resistin increased CXCL8 and CCL2 production by FLSs. Abrogation of CAP1 inhibited the resistin-enhanced CXCL8 production. These results indicate that CAP1 is a functional receptor for resistin on FLSs. CXCL8 has been reported to induce angiogenesis and exerts chemotactic effects on neutrophils and dendritic cells [21, 22]. Furthermore, the inhibition of CXCL8 has been reported to suppress CD14^+^ monocyte-osteoclast differentiation in anti-cyclic citrullinated peptide antibody-positive RA [23]. CXCL8 is strongly expressed in the synovial tissue of patients with RA with a high level of disease activity [24]. Therefore, CXCL8 may be involved in angiogenesis, inflammatory cell migration, and osteoclast differentiation in synovial tissue in RA. CCL2 is also strongly expressed in the synovial tissue [25] and synovial fluid of patients with RA [26]. CCL2 induces the migration and infiltration of monocytes and macrophages [27]. In addition, stimulation with CCL2 enhances the production of IL-6 and CXCL8 by FLSs [28]. CCL2 may be involved in monocyte/macrophage migration and inflammatory molecule production in the synovium in RA. CXCL1 is also increased in the serum, synovial fluid, and synovial tissue of patients with RA [29], and is produced by synovial neutrophils, macrophages, and FLSs [29, 30]. CXCL2 is produced by FLSs in the synovium in RA [31]. CXCL1 and CXCL2 are involved in the migration of neutrophils, proliferation of FLSs, and angiogenesis. CXCL5 is increased in synovial fluid and synovial tissue in RA, and is involved in neutrophil infiltration and angiogenesis [32]. Taken together, resistin contributes to the pathogenesis of RA via chemokine production by FLSs, which may be involved in angiogenesis, inflammatory cell migration, production of inflammatory molecules, and osteoclastogenesis.

In addition, stimulation with resistin upregulated production of IL-6 by RA FLSs. IL-6 might also contribute to chronic inflammation in RA. Increased IL-6 may induce chemokine production by FLSs. However, it is also possible that chemokines upregulated by resistin induced IL-6 production [28].

In the present study, we examined the stimulatory effects of resistin on FLSs. However, macrophages and endothelial cells in the synovial tissue in RA also expressed CAP1. Therefore, further studies are needed in order to elucidate the effects of resistin on macrophages and endothelial cells in RA. We also need to compare resistin and CAP1 expression between early and late RA, and also analyze the effect of treatment to resistin and CAP1 expression to reveal the role of the resistin-CAP1 pathway in the pathogenesis of RA.

## Conclusion

The present results suggest that resistin expressed in synovial tissue in RA contributes to RA pathogenesis by enhancing chemokine production by FLSs in synovial tissue.

## Abbreviations

bp: Base pairs
CAP1: Adenylate cyclase-associated protein 1
CCL: Chemokine (C-C motif) ligand
CXCL: Chemokine (C-X-C motif) ligand
ELISA: Enzyme-linked immunosorbent assay
FBS: Fetal bovine serum
FLSs: Fibroblast-like synoviocytes
GAPDH: Glyceraldehyde-3-phosphate dehydrogenase
IL: Interleukin
mAb: Monoclonal antibody
MMP: Matrix metalloproteinase
OA: Osteoarthritis
pAb: Polyclonal antibody
PCR: Polymerase chain reaction
RA: Rheumatoid arthritis
RNA-Seq: RNA sequencing
ROR1: Receptor tyrosine kinase like orphan receptor 1
RPKM: Reads per kilobase of transcript per million reads mapped
RPMI: Roswell Park Memorial Institute
RT: Reverse transcription
SE: Standard error
siRNA: Small interfering RNA
TLR4: Toll-like receptor 4
TNF: Tumor necrosis factor
vWF: von Willebrand factor
